# Quantification of Finger-Tapping Angle Based on Wearable Sensors

**DOI:** 10.3390/s17020203

**Published:** 2017-01-25

**Authors:** Milica Djurić-Jovičić, Nenad S. Jovičić, Agnes Roby-Brami, Mirjana B. Popović, Vladimir S. Kostić, Antonije R. Djordjević

**Affiliations:** 1 Innovation Center, School of Electrical Engineering, University of Belgrade, Bulevar kralja Aleksandra 73, 11120 Belgrade, Serbia; 2 School of Electrical Engineering, University of Belgrade, Bulevar kralja Aleksandra 73, 11120 Belgrade, Serbia; nenad@etf.rs (N.S.J.); mpo@etf.rs (M.B.P.); edjordja@etf.rs (A.R.D.); 3 Institut des Systèmes Intelligents et de Robotique, Université Pierre et Marie Curie, 4 Place JUSSIEU, 75005 Paris, France; roby-brami@isir.upmc.fr; 4 Institute for Neurology, School of Medicine, University of Belgrade, Dr. Subotića 6, 11120 Belgrade, Serbia; vladimir.s.kostic@gmail.com; 5 Serbian Academy of Sciences and Arts, Knez Mihajlova 35, 11001 Belgrade, Serbia

**Keywords:** finger tapping, angle estimation, inertial sensors, motor assessment, finger aperture

## Abstract

We propose a novel simple method for quantitative and qualitative finger-tapping assessment based on miniature inertial sensors (3D gyroscopes) placed on the thumb and index-finger. We propose a simplified description of the finger tapping by using a single angle, describing rotation around a dominant axis. The method was verified on twelve subjects, who performed various tapping tasks, mimicking impaired patterns. The obtained tapping angles were compared with results of a motion capture camera system, demonstrating excellent accuracy. The root-mean-square (RMS) error between the two sets of data is, on average, below 4°, and the intraclass correlation coefficient is, on average, greater than 0.972. Data obtained by the proposed method may be used together with scores from clinical tests to enable a better diagnostic. Along with hardware simplicity, this makes the proposed method a promising candidate for use in clinical practice. Furthermore, our definition of the tapping angle can be applied to all tapping assessment systems.

## 1. Introduction

During neurological examinations, assessment of motor performance is helpful in identifying the integrity of central nervous system components. Motor tests commonly include speed and strength level evaluation. Finger tapping is a test of the primary motor speed of the index finger [1]. Patients tap fingers as quickly as possible for consecutive 10–15 s trials. The rhythm, amplitude (expressed in terms of either an angle or a distance between fingers), and velocity of the tapping movements vary with patient’s motor capabilities and symptoms.

The tapping test has been widely used for assessment of ataxia [2], stroke recovery [3], Alzheimer’s disease [4], and of bradykinesia in Parkinson’s disease (PD) [5,6,7,8,9]. It is included in the Unified Parkinson’s disease rating scale (MDS-UPDRS test, [10]). In patients with PD, the finger-tapping test was selected because the pattern can be more severely impaired than hand opening and closing or hand pronation and supination elements of the motor section of MDS-UPDRS [11,12,13].

In clinical practice, tapping is often evaluated visually, estimating speed, amplitude, and regularity of movements. However, small amplitude differences cannot be easily and correctly identified. It has been reported that the tapping score was one of the most difficult items to assess [14,15]. Detection of small amplitude differences (among various patients or during follow-up of a patient) can be more precisely performed from numerical data than visually. Differences of several degrees can be identified objectively using an instrumented method, whereas the visual observation is prone to subjective assessment.

Several research groups worked on increasing the accuracy of quantitative evaluation by using various finger-tapping measurement systems [15,16,17,18,19,20,21,22,23,24,25,26,27]. Some relied on 3D recordings from an optical motion capture system with markers placed on the subject’s hands [18,19,20]. In other studies, sensors were mounted on subject’s fingers or constructed as touch pads [21,22,23]. 

In reference [18], an optical motion capture system was used, measuring amplitude, cycle duration, and mean speed of taps. This study showed a difference in tapping patterns between patients with PD and those with progressive supranuclear palsy (PSP). However, optical motion capture systems are expensive, they are demanding for data processing, and require dedicated space for assessment. Additionally, the view of camera markers can be obstructed while patients are performing tapping tasks.

In reference [23], a finger-tapping acceleration measurement system was presented for quantitative diagnosis of PD, based on 3D accelerometers and a pair of touch sensors attached to fingerstalls on the subject’s index finger and thumb. The features included finger-tapping velocities, distance amplitudes, and standard deviation of tapping intervals. However, this method requires strict control of the hand position so that the hand is placed on the desk and finger movements are parallel to the desk.

Another system used a magnetic sensor with two coils mounted on fingers [25]. The coil voltage, due to the electromagnetic induction, depends on the distance between the coils. The system outputs were fingertip distance, velocity, and acceleration. However, this system is prone to errors when the mutual orientation of coils is changed, as this may be interpreted as variations of the distance. The system can be sensitive to the presence of nearby metallic and ferromagnetic objects. 

Systems with gyroscopes have been used for evaluating bradykinesia [5,8,15,16,28] by assessing the speed (or the angular velocity obtained from gyroscopes), rhythm (variation of the angular velocity), and amplitudes of movements (calculated angle excursions). Researchers have been placing inertial sensors on different finger and hand parts to assess bradykinesia and estimate the movement amplitudes. The tapping amplitudes can be quantified through angle excursions [8,15,16,27]. Using inertial sensors, the majority of finger-tapping parameters, such as tapping duration, and opening and closing velocities are relatively simple to be calculated. The tapping amplitude, a spatial parameter describing the tapping performance, can be defined and estimated in different ways, e.g., as the absolute angle from the index finger to an axis [8], or the relative angle excursions the thumb and the index finger [15,16]. However, patients with motor impairments perform the finger-tapping task in very different manners, so that the relative 3D movements of fingers can be challenging to define, measure, and express as a single parameter. 

In this paper, we analyze these challenges and we propose a method for quantification of the finger-tapping angle based on miniature inertial sensors (3D gyroscopes) placed on the index finger and thumb, which could be further used for detailed observation and evaluation (including rhythm, shape, amplitude, regularity, etc.). The system comprises only one pair of inexpensive gyro sensors whose outputs can be recorded and processed with ease, following the algorithms proposed in this paper. Based on our pilot study comprising 37 patients and 12 healthy subjects, we have established that the finger tapping, defined as relative rotational movement of the index finger and thumb, can be considered as a general 3D movement. That movement can include rotations around various axes, but in all cases, there exists a dominant axis of rotation. This axis can vary from subject to subject, also depending on the sensor placement, but it can always be unambiguously found. Therefore, we provide a simplified description of the finger tapping in terms of a single scalar value, which describes the rotation around this dominant axis. This scalar is the angle between the main (longitudinal) axes of the thumb and the index finger, which is also a highly intuitive measure for clinicians. The maximal value of the tapping angle is equivalent to the finger-tapping amplitude, which is typically estimated visually within the MDS-UPDRS scale, part III [10]. We deal with the relative position between the thumb and index finger by obtaining and processing gyro data for both fingers. Unlike techniques that consider movements of only one finger, our method can be used in any environment, it does not require any specific position of patient’s hand, it allows the arm to be moved during tapping, and thereby enables the patient to perform the tapping in his/her natural manner. 

The results provided by the proposed method are compared with results obtained by an optical motion capture system (the reference system). The method was verified on twelve healthy subjects, who imitated impaired patterns identified from the pilot study.

Our system enables objective assessment of the following parameters: tapping duration, angular speed, aperture (the difference between the maximal and the minimal angle within a tap), and various other derived quantities that could be easily exported in a form suitable for interpretation by clinicians.

## 2. Materials and Methods

### 2.1. Pilot Study

A pilot study was conducted on three groups of patients, recruited from the movement disorders outpatient clinic in the Institute of Neurology CCS, School of Medicine, University of Belgrade, Serbia: 15 patients with PD, 12 patients diagnosed with PSP, 10 patients with multiple system atrophy (MSA), and 12 age- and sex-matched healthy subjects with no history of neurological or psychiatric disease. All participants gave informed written consent prior to involvement in the study. 

Subjects were asked to sit comfortably in a chair and place their hand in front of them in the most convenient way. They were instructed to perform a sequence of two tasks within each recording. The first task was to hold fingers closed and still, and then make a circular or zigzag movement in space in order to perform autocalibration. The second task was to repeatedly tap their index finger and thumb for 15 s, performing the tapping as wide and fast as possible [18]. On average, they tried tapping 2–3 times before the setup of the sensors and the recording, to be sure they had understood the task and would perform it according to their motor possibilities. Exaggerating the aperture would reduce the tapping rate; exaggerating the tapping rate would reduce the aperture.

A rest period of one minute was set between sequences. Each sequence began and ended with fingers closed. Six tapping sequences per subject were performed (three with each hand) and video-recorded. 

Observational analysis of 294 recorded tapping sequences (90 sequences for PD, 72 for PSP, 60 for MSA, and 72 for healthy controls) showed a variety of tapping patterns. While the control group showed periodically regular and rhythmical patterns, which differed mainly/only in the speed and aperture, the patients exhibited various irregular movements such as tremor, change of thumb or index orientation at connecting points, skipping/freezing of movement during maximal finger opening or closing, connecting fingers with a strong force, etc. The results for the pilot study are summarized in Table 1, showing counts for typical patterns and tapping events within each patient group.

In order to quantify these tapping patterns in a systematic and comparable way, there was a need to define the angle between the thumb and the index finger as a single scalar quantity that could be always applied, and develop a measurement method that could be used with satisfactory accuracy.

In addition to the patterns shown in Table 1, some patients also performed tapping with wide and slow apertures (MSA: 21 trials), with squeezing fingers while tapping (12 trials within PSP group), and tapping with straight index finger (1 PD and 1 PSP patient). Although they may have important symptoms for clinicians, they do not carry any new information or difficulty for angle estimation that were not previously present in the observed cases from Table 1.

### 2.2. Experimental Setup and Testing Protocol

The large pool of patients from the Neurology Institute (Clinical Centre Serbia, Belgrade, Serbia) allowed a good selection of neurological cases. However, we did not have access there to a laboratory with high-precision measurement equipment, such as a motion capture system, which was available at the Institut des Systèmes Intelligents et de Robotique (ISIR, Université Pierre et Marie Curie, Paris, France). Since it was not feasible to transfer the patients there, nor to organize a new clinical study at the ISIR (Paris, France) in a reasonable time, the validation of the angle estimation was performed on twelve healthy subjects (located in Paris), who emulated various tapping patterns captured in the pilot study. All of the participants gave informed written consent prior to the involvement in the study. 

The experimental protocol was similar as in the pilot study. The subjects were given tasks to perform the tapping in all of the patterns defined in Table 1, sequentially. For the tasks 6–11, the subjects were allowed to practice several times before recording, and they observed the recorded videos of patients performing the tapping, so they could faithfully mimic the patients. 

### 2.3. Instrumentation

The system comprises two sensor control units (SCU), which acquire signals from sensors and wirelessly transmit data to a remote computer, as in [29]. A proprietary wireless communication system covers a radius of 20 m indoors [30].

**Figure 1:**
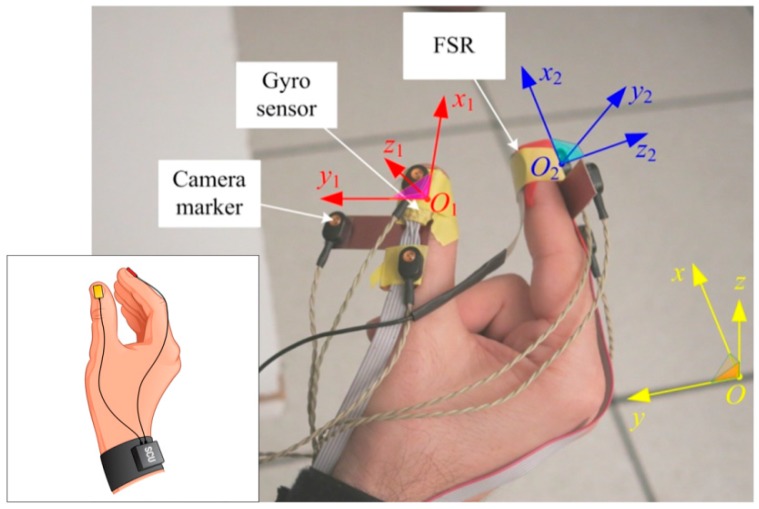
Sketch of the inertial sensor system showing sensors connected to sensor control unit (SCU), and photo of the experimental setup: gyro sensors, camera markers, and FSR sensor mounted on thumb and index finger, global Cartesian coordinate system (*x*,*y*,*z*), and local coordinate systems of the thumb (*x*_1_,*y*_1_,*z*_1_) and index finger (*x*_2_,*y*_2_,*z*_2_).

Three-axial gyro sensors (L3G4200, STMicroelectronics, USA) are mounted on the fingertips of the thumb and index finger, positioned over fingernails, with the *x*-axes of the sensors aligned with the distal segments of the fingers (as shown in Figure 1), and connected to their SCUs (attached to the forearm). In order to measure the contact force between the fingers, a force sensing resistor (FSR, Interlink Electronics, Westlake Village, CA, USA) is connected to a SCU. The sampling rate is 200 samples per second, with 12 bits per sample. The system is small and lightweight, allowing subjects to perform movements freely. The local Cartesian *x*-axis of each gyro sensor is directed along the axis of the finger to which it is attached, the *y*-axis is transversal (Figure 1), and the *z*-axis is perpendicular to the nail and directed outwards.

An optical motion capture system (MOCAP) with active markers (CODA cx1, Charnwood Dynamics Ltd, Rothley, Leicestershire, UK) is used as the reference system for verification of the proposed system. We used the optical motion capture system due to its convenient usage and ability to record general 3D motions with a high resolution (angular resolution 0.002°, lateral position resolution 0.05 mm, and distance resolution 0.3 mm). The disposition of the MOCAP system is shown in Figure 1. Three camera markers are mounted on a small, rigid, T-shaped board and attached to each gyro sensor. Two markers are aligned with the local *x*-axis of the corresponding finger. The third marker is shifted along the *y*-axis.

The acquired signals are monitored online and automatically stored for further processing. The acquisition software was designed in LabWindows CVI (National Instruments, Austin, TX, USA), while the signal analysis was performed in Matlab 7.7.1 (MathWorks Inc., Natick, MA, USA).

### 2.4. Signal Processing

#### 2.4.1. Defining Zero Position

The two gyro sensors measure the vectors of the angular velocities of the thumb and the index finger with respect to the fixed coordinate system (the laboratory). However, the components of these two vectors are obtained in the corresponding local coordinate systems. To estimate the relative movement of the fingers, we need to establish the mutual position (rotation) of the local coordinate systems. Hence, a calibration is performed at the beginning of each recorded sequence. The fingers are pressed against each other so to minimize their relative movement. Thereafter, the whole hand makes a move (e.g., “drawing” one or more circles in the air) during which both sensors measure at least some rotation about all three axes.

Since the relative position of the two sensors is practically fixed, both sensors measure the same vector of angular rotation, $\vec{\omega }={\vec{\omega }}_{1}={\vec{\omega }}_{2}$, where ${\vec{\omega }}_{1}$ is the angular velocity of the thumb, and ${\vec{\omega }}_{2}$ the angular velocity of the index finger. However, the corresponding Cartesian axes of the two sensors are not parallel. Hence, the measured components of the angular velocity ($\omega_{1x},\;\omega_{1y},\;\omega_{1z}$ for the thumb sensor and $\omega_{2x},\;\omega_{2y},\;\omega_{2z}$ for the index-finger sensor) are different.

The rotation (transformation) matrix [R] maps the coordinate system of the thumb into the coordinate system of the index finger [31] (pp. 139–140):

(1) $$\left[\begin{array}{c} \omega_{2x}\\ \omega_{2y}\\ \omega_{2z} \end{array}\right]=\left[\begin{array}{ccc} r_{11} & r_{12} & r_{13}\\ r_{21} & r_{22} & r_{23}\\ r_{31} & r_{32} & r_{33} \end{array}\right]\;\left[\begin{array}{c} \omega_{1x}\\ \omega_{1y}\\ \omega_{1z} \end{array}\right]$$

where $r_{ij},\;i,j=1,\;2,\;3$, are elements of matrix [R]. During the calibration, matrix [R] is nearly time-invariant. The goal of the calibration is to estimate the elements of [R] so to establish the initial relative rotation of the two coordinate systems.

For any time instant during the calibration, we have three relations between each component of ${\vec{\omega }}_{2}$ and three components of ${\vec{\omega }}_{1}$. Each relation involves three elements of [R]. Hence, by knowing the components of ${\vec{\omega }}_{1}$ and ${\vec{\omega }}_{2}$ at three distinct time instants, from Equation (1) we can create three systems of linear equations and compute the elements of [R]. However, these systems of equations can be ill-conditioned. Furthermore, due to various errors (e.g., quantization error and jitter), the resulting rotation matrix may not be orthonormal, requiring further processing (e.g., Gram-Schmidt orthogonalization). An alternative procedure is to use quaternions to relate the two coordinate systems. Instead, we adopted the rotation matrix, because it provides more understandable physical representation of the relative rotation.

To bypass the numerical instabilities and avoid orthogonalization, we evaluate the elements of [R] using an optimization procedure, as follows:

Any rotation of the coordinate system $\left(x_{1},y_{1},z_{1}\right)$ with respect to $\left(x_{2},y_{2},z_{2}\right)$ can be represented as a consequence of three subsequent rotations [31] (p. 151). The first one is the rotation of the first coordinate system about the $x_{2}$-axis of the second system, for $\varphi_{x}$, which is described by the rotation matrix $\left[R_{\varphi_{x}}\right]$. The second one is the rotation about the $y_{2}$-axis, for $\varphi_{y}$, described by $\left[R_{\varphi_{y}}\right]$, and the third rotation is about the $z_{2}$-axis, for $\varphi_{z}$, described by $\left[R_{\varphi_{z}}\right]$. Hence, matrix [R] can be represented as $\left[R]=[R_{\varphi_{z}}][R_{\varphi_{y}}][R_{\varphi_{x}}\right]$ and it is automatically orthonormal. 

With the aim to minimize the differences between the components $\omega_{2x},\;\omega_{2y},\;\omega_{2z}$ and $\omega_{1x},\;\omega_{1y},\;\omega_{1z}$, transformed using Equation (1), we optimize the angles $\varphi_{x},\;\varphi_{y},\;\varphi_{z}$. We construct an optimization function:

(2) $$f(\varphi_{x},\varphi_{y},\varphi_{z})={\left(\omega_{2x}-\left(r_{11}\omega_{1x}+r_{12}\omega_{1y}+r_{13}\omega_{1z}\right)\right)}^{\;2}+{\left(\omega_{2y}-\left(r_{21}\omega_{1x}+r_{22}\omega_{1y}+r_{23}\omega_{1z}\right)\right)}^{\;2}\;+{\left(\omega_{2z}-\left(r_{31}\omega_{1x}+r_{32}\omega_{1y}+r_{33}\omega_{1z}\right)\right)}^{\;2},$$

where the elements of the rotation matrix [R] are functions of the angles $\varphi_{x},\;\varphi_{y},\;\varphi_{z}$, as described in the previous paragraph. We use the nonlinear simplex optimization algorithm to minimize this function and obtain an estimate of $\varphi_{x},\;\varphi_{y},\;\varphi_{z}$ [32]. This method has already been proven useful in gait analysis [33].

Based on the suggested measurement protocol, the autocalibration sequence can be recognized automatically, by identifying the time interval at the beginning of the recording sequence when both sensors register vigorous movements (i.e., large angular velocities), but the magnitudes of the angular velocities are practically equal. 

Matrix [R] evaluated by this calibration is used as the initial rotation matrix for subsequent computations of the tapping angle within one recorded sequence. 

After completing the autocalibration sequence, the tapping commences, when the thumb and the index finger are in relative movement.

#### 2.4.2. Tapping Angle Assessment

In order to assess the tapping angle, we evaluate the relative movement between the thumb and the index finger (the hand may have its own movement, but it is irrelevant). There are various ways to describe this movement. Generally, the movement is complex and requires taking into account all finger joints. It is a combination of rotation and translation and it cannot be exactly described in a way that is useful in clinical practice. To make simplifications, the finger bending is neglected and the movement is assumed to be approximately scissor-like, as suggested in reference [18], where the distance between the finger tips was used as the sole parameter. Along the same guidelines, we describe this relative movement solely in terms of rotation. Such a description is easily obtained from gyroscope sensors and it is simple to evaluate and interpret.

From the recordings, we have found that the index finger has substantially greater swings than the thumb. Additionally, the signal processing is more numerically stable if we observe the movement of the thumb from the index-finger coordinate system. The reason is in the drift that is inherent to the processing of data from gyro sensors. This drift causes errors in the relative rotation of the two local coordinate systems, i.e., it causes errors in matrix [R]. If we observe the rotation from the index-finger coordinate system, the erroneous rotation matrix transforms the relatively small angular velocity of the thumb. If we observe the rotation from the thumb coordinate system, the erroneous rotation matrix transforms the relatively large angular velocity of the index finger, resulting in much larger errors. Hence, we have decided to observe the rotation from the index-finger coordinate system.

The relative rotation of the two fingers can be described in various ways. Generally, three angles are required for a full description. 

**Figure 2:**
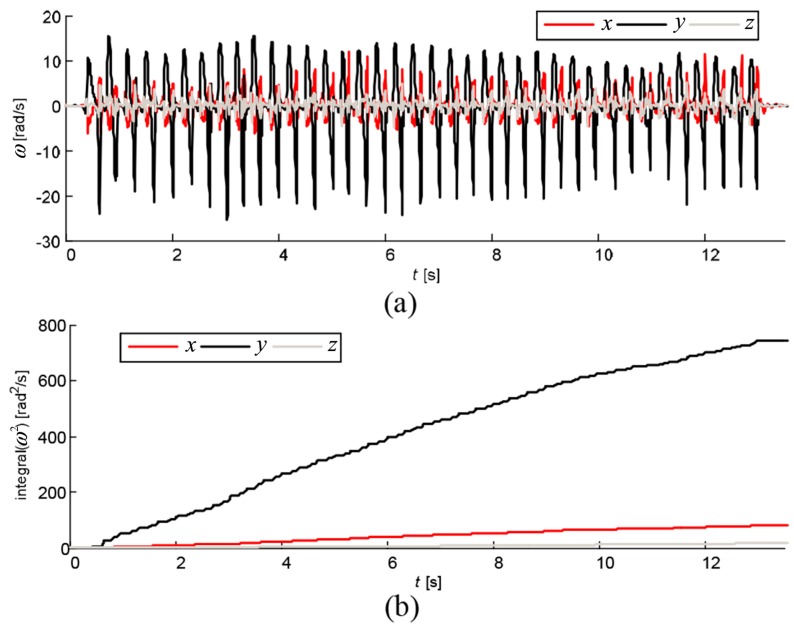
Angular velocity of thumb observed from coordinate system of index finger: (**a**) instantaneous components; and (**b**) integrated squared components.

We observed the relative rotation of the thumb from the coordinate system of the index finger. A typical finger-tapping pattern is shown in Figure 2a. From our pilot study, we have noted that in the majority of the recorded sequences, the dominant component of the relative angular velocity of the thumb is the $y_{2}$-component, i.e., the dominant rotation of the thumb is about the $y_{2}$-axis of the index finger. This can be assessed from the squared components of the angular velocity and their integrals (Figure 2b). By observing the integrals at the end of the recorded sequence, it is clear that the $y_{2}$-component dominates. Averaged over all recorded sequences, the integral of $\omega_{2x}^{2}$ is about 21% of the integral of $\omega_{2y}^{2}$, and the integral of $\omega_{2z}^{2}$ is only 11% of the integral of $\omega_{2y}^{2}$.

In rare cases (around 3% of all observed sequences) when the integral of $\omega_{2y}^{2}$ is not dominant, we computationally rotate the coordinate system of the index finger so that the new $y_{2}$-axis is in the direction of the dominant rotation. In this way, we can cover all tapping patterns. 

The component $\omega_{2y}$ being dominant, the angle of rotation of the thumb about the $y_{2}$-axis of the index finger can serve as a single scalar function that describes the time variations of the tapping angle (the angle between the thumb and the index finger). Having just one function (instead of, for example, three Euler angles) greatly simplifies the tapping quantization [34].

**Figure 3:**
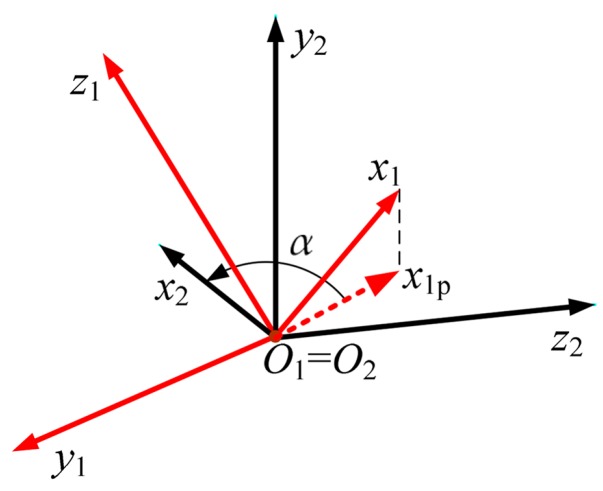
Definition of tapping angle.

We define the tapping angle as follows. First, we project the $x_{1}$-axis onto the $O_{2}x_{2}z_{2}$-plane (Figure 3). We denote this projection by $x_{1p}$. Second, we evaluate the angle *α* (raw tapping angle) between $x_{1p}$ and the $x_{2}$-axis. This is the angle for which $x_{1p}$ should be rotated about the $y_{2}$-axis to coincide with the $x_{2}$-axis. The angle *α* is considered as a signed function of time, α(t), respecting the right-hand rule for the rotation about the $y_{2}$-axis. For the case shown in Figure 3, α>0.

From [31] (p. 139), the projection of the unit vector of the $x_{1}$-axis on the $x_{2}$-axis is $r_{11}$, whereas its projection on the $z_{2}$-axis is $r_{31}$, where $r_{11}$ and $r_{31}$ are elements of the instantaneous rotation matrix [R]. Hence, $\tan\alpha =r_{31}/r_{11}$ and the angle *α* can be evaluated as:

(3) $$\alpha =\mathrm{atan}2(r_{31},r_{11})$$

The function atan2 is built into C and Matlab. Its result is on the semi-closed interval (−π,π].

Generally, the angle α(t) is largest when the fingers touch each other (tapping closure, like in Figure 3). It approaches zero or even becomes negative when the fingers are most widely separated (maximal tapping opening). The minima and maxima differ from subject to subject and even from tap to tap. However, it is more convenient for interpretation by clinicians to have a vanishing tapping angle when the fingers are in contact, while the angle becomes largest when the fingers are fully separated. Therefore, we use another function, θ(t), that defines the tapping angle: we reverse the sign of α(t) and later remove the baseline. We consider the angle θ(t) to be convenient for any assessment system with finger-mounted wearable sensors. Hence, it can be widely used in clinical practice.

The camera system gives the Cartesian coordinates of the markers (with respect to the fixed, laboratory coordinate system). At each time step, from these coordinates we obtain the rotation matrix of the thumb, $\left[R_{1}\right]$, and the rotation matrix for the index finger, $\left[R_{2}\right]$. Both matrices are with respect to the fixed coordinate system. The rotation matrix of the thumb with respect to the index finger is, thereafter, evaluated as $\left[R]={\left[R_{2}\right]}^{-1}[R_{1}\right]$.

#### 2.4.3. Evaluation of Tapping Angle

The first step in the evaluation of the tapping angle from gyro sensors is to compute the relative angular velocity of the thumb with respect to the index finger, ${\vec{\omega }}_{r}={\vec{\omega }}_{1}-{\vec{\omega }}_{2}$. The components of the relative angular velocity in the index-finger coordinate system are:

(4) $$\left[\begin{array}{c} \omega_{rx}\\ \omega_{ry}\\ \omega_{rz} \end{array}\right]=\left[\begin{array}{ccc} r_{11} & r_{12} & r_{13}\\ r_{21} & r_{22} & r_{23}\\ r_{31} & r_{32} & r_{33} \end{array}\right]\;\left[\begin{array}{c} \omega_{1x}\\ \omega_{1y}\\ \omega_{1z} \end{array}\right]-\left[\begin{array}{c} \omega_{2x}\\ \omega_{2y}\\ \omega_{2z} \end{array}\right].$$

During tapping, the two coordinate systems rotate with respect to each other. Hence, the rotation matrix must be updated, using a time-stepping procedure. The updating starts from the calibration sequence. The rotations of the thumb coordinate system about the Cartesian axes of the index-finger system are $\Delta \varphi_{x}=\omega_{rx}\Delta t$, $\Delta \varphi_{y}=\omega_{ry}\Delta t$, $\Delta \varphi_{z}=\omega_{rz}\Delta t$, where Δt=5 ms is the time step. Assuming that these rotations are small, we update the rotation matrix for the next time step as [31] (p. 142):

(5) $$\left[R_{n+1}]=\left[\begin{array}{ccc} \cos\Delta \varphi_{z} & -\sin\Delta \varphi_{z} & 0\\ \sin\Delta \varphi_{z} & \cos\Delta \varphi_{z} & 0\\ 0 & 0 & 1 \end{array}\right]\;\;\left[\begin{array}{ccc} \cos\Delta \varphi_{y} & 0 & \sin\Delta \varphi_{y}\\ 0 & 1 & 0\\ -\sin\Delta \varphi_{y} & 0 & \cos\Delta \varphi_{y} \end{array}\right]\;\;\left[\begin{array}{ccc} 1 & 0 & 0\\ 0 & \cos\Delta \varphi_{x} & -\sin\Delta \varphi_{x}\\ 0 & \sin\Delta \varphi_{x} & \cos\Delta \varphi_{x} \end{array}\right]\;[R_{n}\right]$$

where $\left[R_{n}\right]$ is the rotation matrix at the current time step. Thus, we know the rotation matrix at each time step and evaluate the raw tapping angle from Equation (3). We refer to this technique of angle estimation as the continuous algorithm (AL-C). 

**Figure 4:**
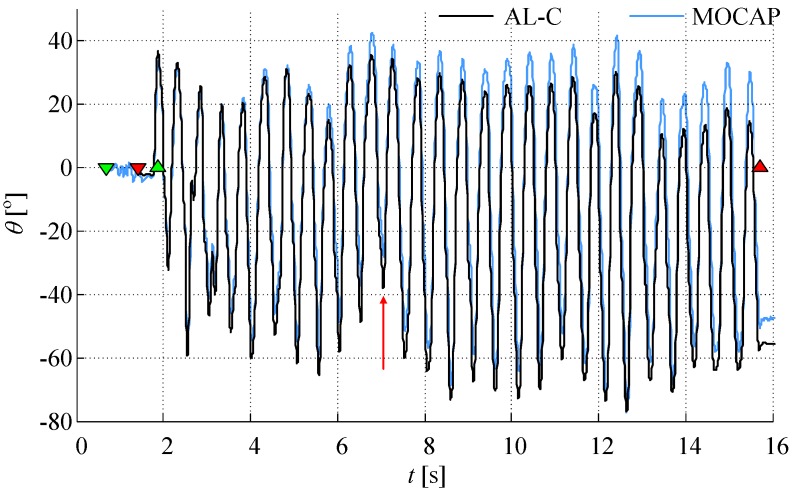
Tapping angle obtained from cameras and gyro sensors with the continuous (AL-C, black line) algorithm (prior to baseline removal), compared to the motion capture system (MOCAP, blue line). The signals are shown for one subject and tapping task #9 (skip tap while fingers open, marked with a vertical red arrow around 7 s). Triangles oriented downward mark the calibration sequence, while upward triangles mark the tapping sequence; start and ending are in green and red colors, respectively.

Figure 4 shows an example of angles obtained from cameras and from AL-C algorithm. The influence of a small drift can be observed at the end of the recorded sequence. The first two markers (downward triangles) denote the beginning and the end of the calibration sequence. The next two markers (upward triangles) denote the beginning and the end of the tapping sequence. The angle obtained from the cameras has an inherent offset due to the initial position of the fingers when the fingers are in contact.

In cases when the drift is large (from our experience, when the final drift exceeds 60°), we can approximately calculate the tapping angle in another way. We have already noted that the dominant rotation of the thumb is with respect to the $y_{2}$-axis of the index finger. Matrix [R] changes as the time lapses. However, we have noted that, in all experiments, its elements in the second row ($r_{21}$, $r_{22}$, and $r_{23}$) remain practically constant. The vector ${\vec{\omega }}_{1}$ has, generally, all three components in the thumb coordinate system, but the coefficients that are used to evaluate the projection of ${\vec{\omega }}_{1}$ onto the $y_{2}$-axis in Equation (4) are practically independent of time. Hence, it is sufficient to know [R] at the beginning of the tapping sequence (as obtained from the calibration) and to project ${\vec{\omega }}_{1}$ on the $y_{2}$-axis. The $y_{2}$-component of the relative angular velocity is:

(6) $$\omega_{ry}\approx r_{21}^{0}\omega_{1x}+r_{22}^{0}\omega_{1y}+r_{23}^{0}\omega_{1z}-\omega_{2y}$$

where $r_{21}^{0}$, $r_{22}^{0}$, and $r_{23}^{0}$ are not updated at all. The tapping angle is, thereafter, evaluated by numerically integrating $\omega_{ry}$ in a time-stepping procedure. This integration is also prone to drift, but it is easily removed by restoring the baseline, as explained in Section 2.4.4. (The AL-C algorithm also requires extraction of the baseline.) We refer to the above described estimation as the resetting algorithm (AL-R) because the rotation matrix is actually reset to its initial value at each time step.

#### 2.4.4. Identification of Individual Taps

The baseline position varies from tap to tap because the taps are not uniform and due to the drift associated with the processing of gyro signals. Hence, we have developed and implemented an algorithm for the baseline removal. The main task is to identify starting and ending time instants of individual taps. The algorithm includes finding the average tapping period using the autocorrelation of θ(t), filtering by averaging (to remove noise due to vibrations, bumps when the fingers touch each other, slow and non-decisive changes of the direction of finger movement, sensor noise, etc.), and finding minima and maxima of the filtered function. The points of minima represent the starting and ending points of individual taps. The average tapping period is used as an educated initial guess for a local heuristic search aimed at separating two local minima of θ(t).

**Figure 5:**
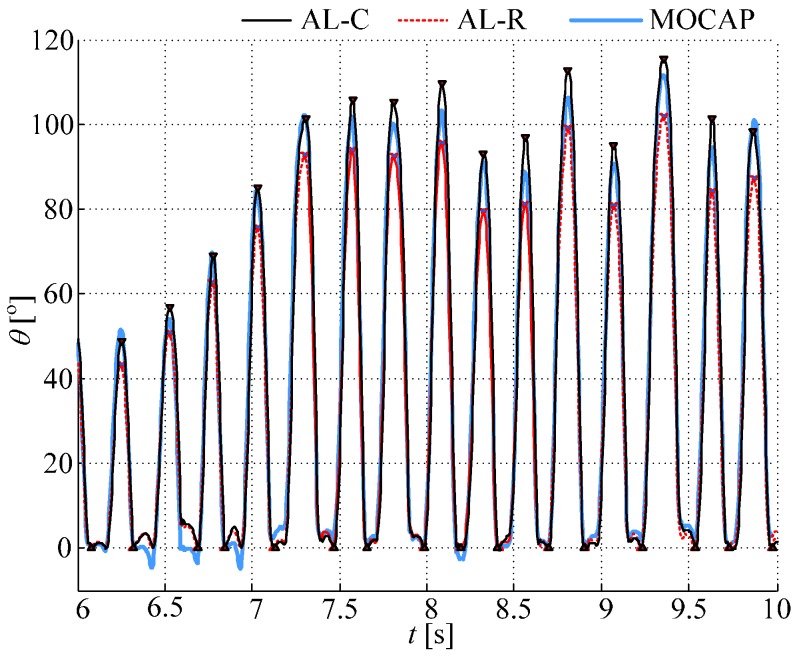
Tapping angle obtained from gyro sensors (with removed baseline) using the continuous (AL-C, black solid line) and resetting (AL-R, red dotted line) algorithms, and angle obtained from motion capture system (MOCAP, blue solid line). Shown is an example for one subject performing task #2 (fast and wide).

Figure 5 shows an example of the function θ(t) with removed baseline, for continuous and resetting algorithms, overlapped with the angle obtained from cameras for one subject performing tapping task 2. For the camera angle, the baseline is not removed; just a constant offset is added so that the angle equals zero at the beginning of the tapping sequence. The triangular red markers show estimated angle minima and maxima.

## 3. Results

**Figure 6:**
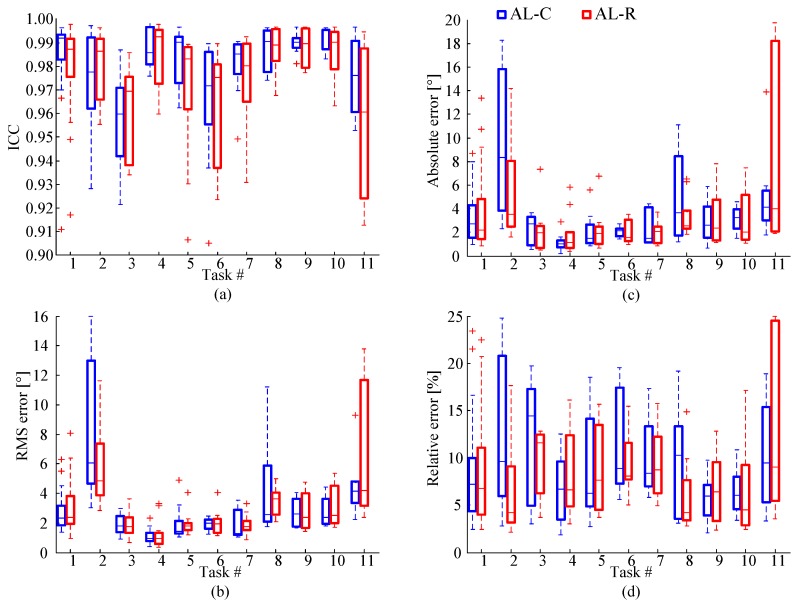
Boxplots comparing tapping angles obtained from gyro sensors and cameras in terms of: (**a**) Intraclass correlation coefficient (ICC); (**b**) RMS error between angles; (**c**) absolute error for tapping aperture; and (**d**) relative error for tapping aperture. Results are presented for all subjects, grouped according to eleven tapping tasks from Table 1, showing average values and standard deviations, for continuous and resetting algorithms.

We compare angles obtained by the proposed method with angles obtained from the camera system using the following error measures: the intraclass correlation coefficient (ICC) [35], the absolute and relative errors in tapping apertures, and the RMS error, as shown in Figure 6.

ICC is evaluated for each tap, and then averaged for the whole recorded sequence. The results for all subjects are shown in Figure 6a, for AL-C and AL-R algorithms, grouped according to the tapping task. For AL-C, the average ICC for all recorded sequences is 0.980. For AL-R, the average ICC is 0.972. These numbers verify an excellent correlation between the angles obtained by the proposed algorithms from the gyro sensors and the angles obtained from the cameras.

As the second error measure, we evaluate the root-mean-square (RMS) error (Figure 6b). The average error for all taps and all recorded sequences is 3.98° for AL-C and 3.16° for AL-R.

Finally, we compare apertures. The absolute and relative errors for the apertures are evaluated for each tap, and then averaged for the recorded sequence (Figure 6c,d). The average absolute error for all recorded sequences is 3.43° for AL-C and 3.18° for AL-R. The corresponding average relative errors are 0.096 and 0.090, respectively. 

The results obtained by the proposed method show a high correlation with those provided by the reference optical motion capture system, proving this method to be usable for clinical practice.

The accuracy of AL-C algorithm was found to be reduced in cases of extremely energetic tapping (task 2), when the gyro sensors approached saturation. Due to these nonlinearities, drift increases, which tumbles the transformation matrix after several seconds. The accuracy of AL-R algorithm is reduced when the fingers slip during tapping or the tapping mode is changed within a recorded sequence (in particular, task 11).

AL-C algorithm naturally follows the relative rotation of the coordinate systems and, hence, gives results close to the actual tapping angles. However, it is prone to drift, which occurs when the tapping is vigorous. In contrast to AL-C, AL-R algorithm is only approximate because it does not allow the relative rotation of the coordinate systems. In most cases, AL-R yields somewhat smaller tapping apertures than AL-C, but it is not prone to drift.

## 4. Discussion

In this paper we provide quantification of the finger-tapping angle based on 3D motion of the thumb and index finger. In spite of the complexity of the motion, we have managed to define the tapping angle as a single scalar quantity.

The study highlights the importance of identifying the dominant tapping axis. The proposed method offers a simple procedure that allows autocalibration, i.e., defining the initial conditions for angle estimation, regardless of hand position and orientation. Although gyroscopes are frequently used for estimation of upper and lower limb angles and there are papers describing solutions that performed estimation of the finger-tapping amplitude, these issues have not been previously addressed. Additionally, the assessment of various tapping patterns, provoked by motor impairments of the patients*,* has not been covered in the literature.

In clinical applications, the procedure we suggest requires from 2 to 5 min for the complete system setup, out of which less than one minute is needed for mounting the sensors on a patient. Explanations and practicing the tapping require additional 2 min; the same time it takes for the standard clinical validation within the UPDRS test. The autocalibration is performed within each tapping sequence, and it lasts only a few seconds (usually around 2 s), while the tapping sequence is recorded for 15 s. The overall duration for one patient, including mounting the system, explaining the procedure, and recording both hands (each hand repeated three times with one-minute minimal pause between the sequences) is estimated to be between 15 and 20 min.

The post-processing can be performed automatically or manually. For the automatic post-processing, all six sequences from one patient can be processed and parameters extracted in several seconds. For severely impaired patients or specific tapping patterns, one could be advised to perform manual post-processing which would include careful inspection if all taps were correctly identified and correction of the identified taps by adjusting the thresholds, which would increase the post-processing time to 5–7 min per patient.

The system is inherently reusable and the vital components do not suffer degradation of their characteristics over usage in a long period of the time. The methodology we propose can be implemented with any commercially available inertial system. However, different producers offer different qualities of sensors, not only in terms of their sensitivity and resolution, but also in terms of the stability of calibration. Hence, we would advise periodic sensor checkups of signal quality and calibration coefficients. After purchasing the system, the estimated costs are related mainly to the time spent in the implementation of the setup and the measurement procedure, which is of the order of 10 min, with additional costs due to the periodic maintenance of the sensor system.

By comparing tapping angles obtained by our method (using gyro sensors) with results obtained by the reference camera system for various tapping patterns (defined based on a preliminary clinical study), we have verified and validated that the proposed angle calculation can be used for various tapping patterns, from slow and very weak movements to fast and vigorous, for patterns affected by tremor, as well as patterns that comprise irregular tapping movements within a tapping sequence (e.g., skipping a tap or freezing the fingers at the maximal/minimal aperture). Our method is validated for 15 s tapping sequences, as one of the standard assessment protocols performed in clinical settings. However, we have verified by experiments that this method could be applied for longer sequences (e.g., 30 s). Auto-calibration should be performed just before the beginning of the tapping sequence. Although the finger tapping is a repetitive and often a rhythmical task, it can comprise changes in hand and finger orientation during a tapping sequence. Hence, the angle estimation method should be immune to these changes. Based on our pilot study, we were able to record various possible tapping patterns, which enabled us to set specifications for angle calculation algorithms.

We introduced two angle calculation algorithms: continuous and resetting, and compared their results for various tapping patterns. The continuous algorithm can be prone to drift. For tapping patterns with changes in finger orientation during tapping (or at finger closing) the resetting algorithm may lose some information, thus increasing the error and leading to worse results than having a small influence of the drift (Figure 6, tasks 4, 9, 10, and 11). Furthermore, since the tapping angle is evaluated by post-processing, it is easy to a posteriori establish the presence of drift (by observing Figure 4, prior to the removal of the baseline). If a large drift is established, then the results of AL-R can be automatically selected as the more accurate ones.

Our method also includes an algorithm for tapping segmentation, i.e., identification of individual taps. Using the autocorrelation for calculating the average tapping period and following the procedure described in Section 2.4.4, we obtained correct segmentation for all recorded files, even for those with severely impaired taps.

One of the issues that needs to be addressed is the definition of the tapping angle and its justification. As fingers comprise several joints, one can question defining the tapping angle as the angle between the tip segments of the fingers (distal phalanges) without considering other finger segments. Although placing sensors on each finger segment would be the most accurate and precise assessment, as in references [27,36], this is neither practical for clinical assessment, nor for interpretation of the tapping results. In addition to analyzing the diversities of tapping patterns performed by the patients with motor impairments, our goal was to offer a minimal set of sensors and maximal information for clinicians. Several authors performed tapping assessment with only one sensor placed on the index finger and calculated the angle of the finger relative to the vertical axis [8,17,37,38] or, having the hand placed on a table, defined the zero-angle when the finger is down [39]. Although the majority of movement in finger tapping is performed by the index finger, we have found that it was important to also have a sensor on the thumb. First, in order to make finger-tapping assessment from both fingers, and second, to have the relative angle between two fingers, independent on hand orientation and its changes during tapping. This is important for obtaining natural tapping patterns for standardized clinical assessment (e.g., part of UPDRS testing).

Additionally, considering the number of sensors, there is also a question where to place them in order to obtain full tapping assessment based on one angle. Literature offers fitting one angle based on all finger segments [18], calculating the angle based on proximal phalanges [8,39], intermediate phalanges [36,37], and distal phalanges [16,20,22,23,25,40]. Our proposal for assessment of patients with upper limb motor impairments is to place sensors on the distal phalanges, in order to record subtle characteristics in their tapping patterns (such as hesitation while opening/closing and change of connecting point between the fingers at closing).

Based on the proposed measurement system and the angle assessment methodology, the clinicians can be provided with numerical values describing the patient’s tapping performance: tapping duration, angular speed, maximal aperture (the difference between the maximal and the minimal angle within a tap), as well as their deviations and trends within the sequence [41]. This can provide an objective follow up of the patient’s state during routine examinations. For more advanced analysis or research studies, an angle estimation, such as shown in Figure 5, could reveal more information (e.g., quantify freezing and other atypical events). This could be used either by a clinician trained to interpret signals, or by an engineer/technician participating in the team.

By using instrumentation, instead only visual observation, finger-tapping movements can be studied in more detail, including quantification of hypokinesia and/or bradykinesia, detection of start hesitations, freezing episodes, tremor, and other atypical movements.

The benefit of the proposed system (as well as any instrumentation device that could be used for this purpose) is that it can provide the same parameters that clinicians are trained to observe during visual examination when they assign MDS-UPDRS III score (or similar test). These results can be used for automatic quantification of motor performance [42,43].

## 5. Conclusions

The definition and evaluation of the tapping angle that we propose requires only one pair of miniature gyro sensors, placed on the fingertips. This setup enables finger-tapping assessment for tapping patterns that include slow and fast tapping patterns, having small or large tapping apertures, changing hand orientation while tapping, as well as having occasional irregular tapping events, such as skipping taps and other events listed in Table 1.

Our study provides recommendations for tapping assessment procedure, suggesting an autocalibration sequence before the tapping sequence, angle estimation based on an identified dominant tapping axis, and calculation of the tapping angle based on 3D motion of both fingers. As with all angle estimations based on inertial sensors, drift is an important issue. To cope with it, we have designed an algorithm that involves resetting of the rotation matrix. We have considered pros and cons of this algorithm in comparison with an algorithm without resetting.

The proposed assessment method can be used in clinical environment. It uses an affordable sensor system and the procedure is not time or skill demanding. The potential of such a light and inexpensive wearable system is to be used concurrently with scores from clinical tests, providing possibilities for diagnostic support and therapy follow-up.

In the future, we hope to validate these results on patients, also including other patient groups (with motor impairments). We wish to perfect this system so that it could be adopted by clinicians in their everyday work, as well as by other research groups interested in such assessment.

## Figures and Tables

**Table 1:** Typical tapping patterns and events identified from the pilot study, and their numbers of occurrences within the observed patient groups.

#	Tapping Pattern	PD	PSP	MSA
1	Tap moderately fast and wide	30	18	6
2	Fast and wide	12	6	3
3	Fast but with small apertures	24	21	13
4	Slow with small apertures	24	24	17
5	Change hand rotation while tapping	/	3	/
6	Tremor	6	2	4
7	Change thumb orientation while tapping	8	13	/
8	Make strong impacts while tapping	18	11	1
9	Occasionally skip a tap by holding fingers open	8	9	11
10	Occasionally skip a tap by holding fingers nearly closed	/	5	/
11	Tap and slip the thumb over the index finger	6	5	3
